# Differential Regulation of Circulating Soluble Receptor for Advanced Glycation End Products (sRAGEs) and Its Ligands S100A8/A9 Four Weeks Post an Exercise Intervention in a Cohort of Young Army Recruits

**DOI:** 10.3390/biom11091354

**Published:** 2021-09-13

**Authors:** Ioannis-Alexandros Drosatos, James N. Tsoporis, Shehla Izhar, Sahil Gupta, George Tsirebolos, Eleftherios Sakadakis, Andreas S. Triantafyllis, Angelos Rigopoulos, Dimitrios Rigopoulos, Loukianos S. Rallidis, Ioannis Rizos, Thomas G. Parker

**Affiliations:** 1 Second Department of Cardiology, Attikon University Hospital, 12462 Athens, Greece; ioannisdrosatos@gmail.com (I.-A.D.); gtsirebolos@icloud.com (G.T.); elsakadakis@yahoo.gr (E.S.); andtridoc@yahoo.gr (A.S.T.); angelos.rigopoulos@gmail.com (A.R.); lrallidis@gmail.com (L.S.R.); ioannis.c.rizos@otenet.gr (I.R.); 2 Department of Preventive Medicine, 414 Military Hospital, 15236 Athens, Greece; dr.rigopoulos@gmail.com; 3 Keenan Research Centre for Biomedical Science, Li Ka Shing Knowledge Institute, Unity Health Toronto, University of Toronto, Toronto, ON M5B 1W8, Canada; shehla.izhar@unityhealth.to (S.I.); sahil.gupta@unityhealth.to (S.G.); thomas.parker@unityhealth.to (T.G.P.); 4 Department of Cardiology, 401 General Military Hospital of Athens, 11525 Athens, Greece

**Keywords:** exercise, physical activity, inflammation, sRAGEs, S100A8/A9, IL-6

## Abstract

Apart from its beneficial effects on cardiovascular risk factors, an anti-inflammatory effect of exercise is strongly implicated. Yet, data regarding the effect of an exercise intervention on healthy individuals are limited and contradictory. The present study aimed to investigate the effects of a physical activity intervention on the soluble form of the receptor for advanced glycation end products (sRAGEs) and its ligands S100A8/A9. A total of 332 young army recruits volunteered and 169 completed the study. The participants underwent the standard basic training of Greek army recruits. IL-6, IL-1β, S100A8/A9, and sRAGEs were measured at the beginning and at the end of the training period. Primary rodent adult aortic smooth muscle cells (ASMCs) were analyzed for responsiveness to direct stimulation with S100A8/A9 alone or in combination with sRAGEs. At the end of the training period, we observed a statistically significant reduction in S100A8/A9 (630.98 vs. 472.12 ng/mL, *p* = 0.001), IL-1β (9.39 [3.8, 44.14] vs. 5.03 [2.44, 27.3] vs. pg/mL, *p* = 0.001), and sRAGEs (398.38 vs. 220.1 pg/mL, *p* = 0.001). IL-6 values did not change significantly after exercise. S100A8/A9 reduction was positively correlated with body weight (r = 0.236 [0.095, 0.370], *p* = 0.002) and BMI (r = 0.221 [0.092, 0.346], *p* = 0.004). Direct stimulation of ASMCs with S100A8/A9 increased the expression of IL-6, IL-1β, and TNF-α and, in the presence of sRAGEs, demonstrated a dose-dependent inhibition. A 4-week military training resulted in significant reduction in the pro-inflammatory cytokines IL-1β and S100A8/A9 complex. The observed reduction in sRAGEs may possibly reflect diminished RAGE axis activation. Altogether, our findings support the anti-inflammatory properties of physical activity.

## 1. Introduction

Regular physical activity is considered a cornerstone of cardiovascular disease (CVD) prevention and is, therefore, strongly encouraged by most medical societies [1]. Its beneficial effects on classic cardiovascular (CV) risk factors such as hypertension, high-density lipoprotein, obesity, and insulin sensitivity are well established. Additionally, there is biological plausibility that exercise may have anti-inflammatory effects [2]. Chronic low-grade inflammation is a recognized risk marker of CVD [3]. There is a wealth of epidemiological data and observational studies supporting the anti-inflammatory properties of exercise, as illustrated by an inverse relationship between physical activity and classic inflammatory markers [4]. However, even in those studies, the effect varied according to exercise type and intensity [5].

Data from interventional trials regarding the effects of physical activity on inflammation in apparently healthy individuals are limited, with C-reactive protein (CRP) and intereleukin-6 (IL-6) being the most thoroughly studied markers. The few relatively large studies (*n* > 100 in the intervention group) have provided contradicting results [6,7,8,9]. Moreover, smaller studies exhibited heterogeneous results when two or more inflammatory markers were tested, implying different sensitivity and/or different modes of reaction of each biomarker to exercise [10,11]. IL-1β is a key regulator of inflammatory processes [12] and its blockade has been proposed as a potential treatment for a variety of chronic diseases. IL-1β’s response to exercise has not been extensively investigated, with some studies reporting a reduction and others finding no significant effect [13]. More recently, several novel markers of the inflammatory process have been identified.

Advanced glycation end products (AGEs) and their cellular receptors (Receptor of Advanced Glycation End Products, RAGEs) are associated with chronic disease and, especially, long-standing inflammation. Soluble RAGEs (sRAGEs) are produced through two different mechanisms: by proteolytic cleavage of the extracellular domain of RAGE, or by alternate mRNA splicing and termed endogenous secretory RAGEs (esRAGEs). esRAGEs comprise, on average, 20–25% of total sRAGEs [14]. The prevailing hypothesis suggests that sRAGE functions as a decoy, binding RAGE ligands (e.g., AGEs, S100 proteins, High Mobility Group Box-1 (HMGB-1)) in plasma and therefore preventing the downstream inflammatory cascade. This hypothesis is supported by animal studies, where injection of sRAGEs exhibited anti-inflammatory effects [14]. Interestingly, human studies on sRAGEs have not been consistent. In many studies, low levels of sRAGEs were related with the disease state (chronic obstructive pulmonary disease, cancer, atherosclerosis) [15,16,17]. Conversely, several studies have reported a positive correlation of sRAGEs with known pro-inflammatory markers and adverse outcomes [18,19,20,21,22]. Interestingly, a relative homogeneity of results is observed in patients with diabetes, who exhibit higher sRAGEs values compared to healthy controls in most studies [23,24].

S100A8 and S100A9 are low molecular weight proteins, consisting of 93 and 114 amino acids, respectively, and belong to the S100 proteins superfamily [25]. Due to their calcium-binding properties and high expression levels in granulocytes, they are also termed Calgranulin A and Calgranulin B [25]. They exist mostly in the form of heterodimer due to stability reasons. S100A8/A9 is actively secreted from myeloid cells during inflammatory processes and serves to mediate immune responses. It has also been characterized as damage-associated molecular pattern molecule (DAMP). It exerts its functions via binding to RAGE and/or TLR4 (Toll-Like Receptor 4) [25]. S100A8/A9 has been associated with inflammatory diseases and response to treatment [26,27]. In healthy individuals, high plasma levels of S100A8/A9 predict cardiovascular events and adverse outcomes in heart failure patients [28,29]. Furthermore, S100A8/A9 levels correlate positively with the severity of disease in coronary artery patients [30,31]. S100A8/A9 may therefore serve not only as a biomarker for diagnosis and prognosis of inflammation-associated diseases but as an indicator of response to therapeutic treatments as well. 

Data regarding the impact of physical activity on the above-mentioned molecules are conflicting (sRAGEs) or sparse (S100A8/A9). Thus, the aim of our study is to examine the impact of an exercise intervention on the aforementioned markers in a relatively homogeneous sample of young army recruits in comparison with the effects on well-established markers such as IL-1β and IL-6.

## 2. Methods

### 2.1. Participants

Four hundred forty-seven (447) young recruits in a Greek military training camp were asked to participate in our study. Medical history was obtained from the three hundred ninety-eight (398) who volunteered to assess eligibility. Inclusion criteria were: freedom from any acute or chronic disease and no medication use. Three hundred thirty-two (332) participants met the inclusion criteria. All subjects were male. Blood pressure was measured twice using a manual sphygmomanometer (Rudolf Riester GmbH, Jungingen, Germany) after 10 min rest and the mean value was recorded. Body weight was assessed to the nearest 0.1 kg using an electronic scale. A stadiometer was used to measure height without shoes to the nearest cm. BMI was calculated as the weight in kilograms divided by height in meters squared. Anthropometric characteristics are shown in Table 1. One hundred sixty-three (163) participants did not complete the study for various reasons (withdrawal of consent, acute disease/injury, failure to complete) so the final sample consisted of one hundred sixty-nine (169) participants. Informed consent was obtained from all participants before inclusion. Study protocol was approved by the Ethical Commission of Attikon University Hospital as well as by the Greek Army Health Agency.

### 2.2. Intervention

The study had a pretest–posttest one group quasi-experimental design. The participants were housed for the entire period and were offered identical food choices. Physical intervention was standardized for all participants according to Greek army training protocol. Training during the first week included 7 h of moderate-intensity activities (drills, brisk walking, or marching under load) and 2 h of moderate-to-high-intensity aerobic exercise (running), while during weeks two to four, the participants underwent 6 h of moderate-intensity activities and 3 h of moderate-to-high-intensity aerobic exercise. Exercise sessions were equally distributed across weekdays with one day rest on Sundays.

### 2.3. Blood Samples

Blood samples were collected from all participants in a non-fasting state at baseline and at the end of the training period. They were immediately allocated in EDTA containers, centrifuged at 3500 rpm for 10 min and stored at −80 °C until analysis. The plasma concentrations of S100A8/S100A9, sRAGE, IL-1β, and IL-6 were quantified using the human S100A8/S100A9 heterodimer (sensitivity—21.5 pg/mL), RAGE (sensitivity—16.14 pg/mL), IL-1β (sensitivity—3.9 pg/mL), and IL-6 (sensitivity—0.7 pg/mL) DuoSet ELISA kits, respectively, according to the manufacturer’s instructions (R&D Systems Inc., Minneapolis, MN, USA).

### 2.4. Cell Culture

All animal experiments conformed to protocols approved by St. Michael’s Hospital Animal Care Committee in accordance with the Guide for the Care and Use of Laboratory Animals. Male Sprague–Dawley rats weighing 200–225 g were sacrificed and the ascending thoracic aorta (above the heart to the left of the subclavian artery) was aseptically excised. Excised aortic tissue was placed in Hanks’ balanced salt solution (HBSS, Gibco, Grand Island, NY, USA) and cultured as previously described [32,33]. In brief, adhering fat and connective tissue were removed by blunt dissection. Vessels were then opened longitudinally and preincubated in HBSS containing 1 mg/mL collagenase (type C LS II, 146 U/mg, Worthington Biochemical Corp., Freehold, NJ, USA) and 0.5 mg/mL elastase (type I, 32 U/mg, Sigma, St. Louis, MO, USA). The adventitia was carefully removed. After dissection, aortas were placed in a fresh enzyme solution, minced into 1-mm pieces, and incubated for an additional 1.5–2.0 h with trituration at 30 min intervals. Cells were then seeded at a density of 1.0 × 10^4^ viable cells cm^−2^ in plastic culture dishes. Cells were harvested for passaging at 2 wk intervals with a trypsin- (0.01%, VMF, Worthington Biochemical Corp., Freehold, NJ, USA) EDTA (0.02%, Sigma, St. Louis, MO, USA) solution and plated. Passaged cells were grown in either DMEM containing 10% FCS plus antibiotics or in serum-free medium with or without 10% FCS supplementation depending on the experiment. Confluent cells were used after 6 days in primary culture. Cells grew in the typical hill and valley pattern characteristic of ASMCs and formed multiple cell layers based on both light (Olympus Upright BX50, Cansen Group, Markham, ON, Canada) and electron microscopic (T20 TEM, Thermo Fischer, Waltham, MA, USA) examination. For in vitro experiments, the *n* represents the number of separate experiments. Within each experiment there was a minimum of 3 replicates. 

### 2.5. ASMC Treatment

ASMCs were treated with vehicle (PBS), recombinant human (S100A8 (0.5 or 0.05 µg/mL), S100A9 (0.5 or 0.05 µg/mL)) (R&D Systems, Minneapolis, MN, USA) and sRAGE (0.5 or 0.05 ng/mL) (Prospec, Ness-Ziona, Israel) alone for 1 h or in combination for 24 h.

### 2.6. Real Time RTPCR

RNA was harvested from crushed aortic tissue or adherent ASMCs using the RNeasy Plus mini kit (Qiagen, Alameda, CA, USA) according to the company’s protocol. Real-time quantitative RT-PCR was performed according to the instructions of the manufacturer (Qiagen, Alameda, CA, USA) [34]. In brief, a 25 µL reaction volume containing 12.5 µL of RT^2^ SYBR Green qPCR Master Mix, 10.5 µL of H_2_O, 1 µL of gene-specific 10 µM PCR primer-pair stock of gene of interest (rat and human primers for 18S, HSP70, Bax, and Bcl2-proprietary sequences) (Qiagen, Alameda, CA, USA), and 1 µL of RT^2^ First-Strand cDNA (template) underwent a two-step cycling program: 40 cycles, 10 min at 95 °C, 15 s at 95 °C, and 1 min at 60 °C. In separate experiments, the threshold cycle (Ct) value for the housekeeping gene, 18S, and for the gene of interest in each sample was determined. For each sample, the difference between the C_t_ values (ΔCt) for each gene of interest and 18S was calculated. For each pair-wise set of samples to be compared, the difference in ΔCt values (ΔΔC_t_) for the genes of interest between the two samples were calculated. Due to the guaranteed consistently high levels of amplification efficiency across the RT-2 qPCR Primer Assays, the fold-change in gene expression is equal to 2^(−Δ^^ΔCt)^. For each gene of interest in the experimental group, the mean fold-change is shown relative to the gene of interest in the control group.

### 2.7. Statistical Analysis

Statistical analysis was completed using SPSS package for Windows (Version 20.0, IBM SPSS, Markham, ON, Canada). S100A8/A9 and sRAGE values were normally distributed and are presented as mean ± standard deviation (SD). Because of their non-normal distribution, IL-1β and IL-6 values are presented as median value and interquartile range. Correlations involving normally distributed data were calculated using Pearson’s correlation, whereas Spearman’s rho was used for non-parametric data. We used paired samples *t*-test to assess the effect of exercise on sRAGEs and S100A8/A9 while Wilcoxon test was used for IL-1β and IL-6. Subgroup effects according to BMI class and baseline biomarkers values were assessed using one way ANOVA (Version 20.0, IBM SPSS, Markham, ON, Canada). A 2-sided *p*-value of less than 0.05 was considered statistically significant.

## 3. Results

**Figure 1:**
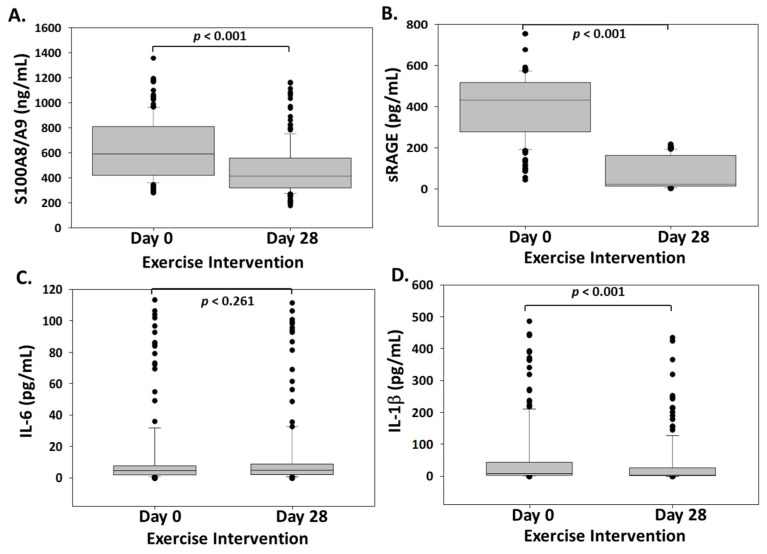
Boxplot chart showing the changes in plasma S100A8/A9 (ng/mL) (**A**), sRAGE (pg/mL) (**B**), IL-6 (pg/mL) (**C**), and IL-1β (pg/mL) (**D**) presented as box plots showing median and interquartile range. *n* = 169.

After completion of 4 weeks of military training, a significant effect on S100A8/A9 and sRAGE was observed (Figure 1). S100A8/A9 levels were reduced from 630.98 ± 239.75 ng/mL at baseline to 472.12 ± 207.19 ng/mL (*p* < 0.001) at the end of training. Soluble RAGEs were also reduced from 398.38 ± 148.9 pg/mL to 220.1 ± 180.31 pg/mL (*p* < 0.001). IL-1β was also significantly reduced (9.39 pg/mL [3.8, 44.64] to 5.03 pg/mL [2.44, 27.3]). On the other hand, no significant difference was observed between IL-6 values at baseline (4.76 pg/mL, [2.15, 7.79]) and at the end of the training period (5.27 pg/mL, [2.34, 9.04]).

Baseline values of IL-6 and S100A8/A9 were significantly correlated with baseline IL-1β (IL-6: r = 0.873, *p* < 0.001, S100A8/A9: r = 0.163, *p* = 0.034). Likewise, a reduction in IL-1β after exercise was positively correlated with baseline IL-6 (r = 0.646, *p* < 0.001).

**Figure 2:**
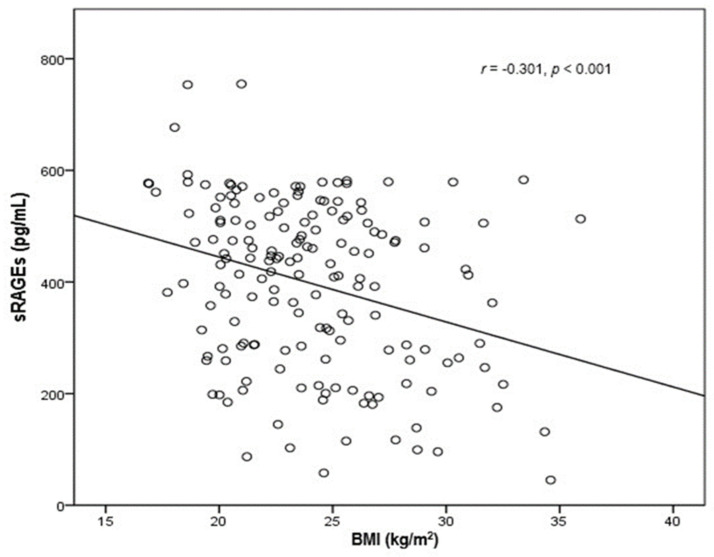
Scatterplot graph showing a negative relationship between baseline sRAGE levels and BMI. *n* = 166.

**Figure 3:**
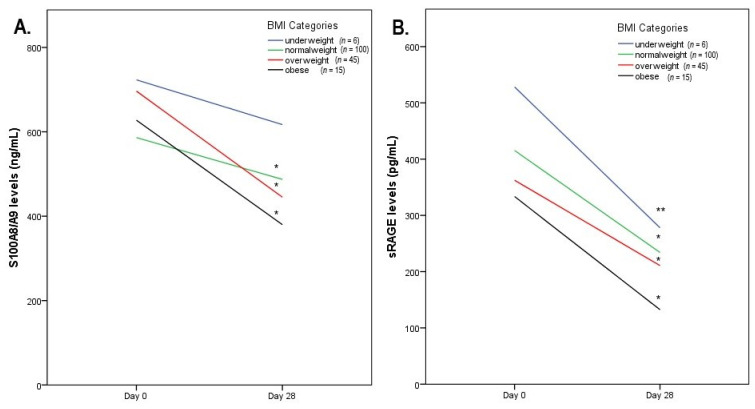
Line charts with biomarkers’ changes across BMI categories for S100A8/A9. (**A**) * *p* < 0.001 vs. baseline; sRAGE (**B**) * *p* < 0.001 vs. baseline, ** *p* < 0.005 vs. baseline. BMI Categories according to WHO Classification: <18.5 underweight, 18.5–25 normal weight, 25–30 overweight, >30 obese.

After excluding three individuals for whom BMI data were missing, we additionally evaluated the correlations of the investigated biomarkers with BMI. Baseline sRAGEs were significantly and inversely correlated with BMI (r = −0.301, [−0.445, −0.141], *p* < 0.001) (Figure 2), whereas the reduction in S100A8/A9 after the exercise intervention was positively correlated with body weight (r = 0.236 [0.095, 0.370], *p* = 0.002) and BMI (r = 0.221 [0.092, 0.346], *p* = 0.004). We further stratified the participants into categories, at first according to the median BMI (24.035) and then according to World Health Organization (WHO) established BMI categories. Although most of the subgroups (apart from underweights for S100A8/A9) demonstrated statistically significant changes (Table 2 and Table 3), we observed a distinct pattern in the change in each biomarker according to BMI categories (Figure 3). Interestingly, sRAGEs were reduced similarly across all BMI subgroups while S100A8/A9 demonstrated a sharper decrease in obese and overweight patients.

**Figure 4:**
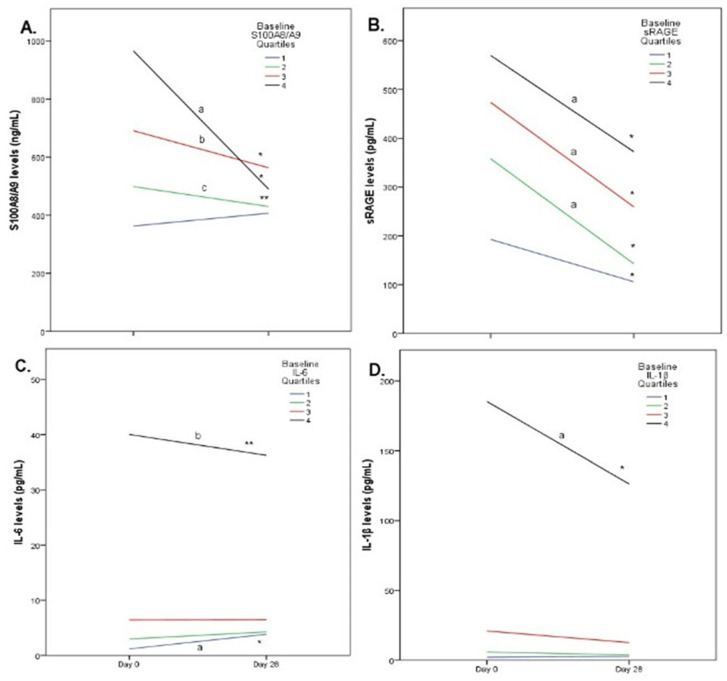
Line charts with biomarkers’ changes according to baseline values for S100A8/A9. (**A**) * *p* < 0.001 vs. baseline, ** *p* < 0.01 vs. baseline, **^a^** *p* < 0.001 vs. 1st, 2nd and 3rd quartile, ^b^ *p* < 0.001 vs. 1st quartile, **^c^** *p* < 0.01 vs. 1st quartile; sRAGE (**B**) * *p* < 0.001 vs. baseline, **^a^** *p*< 0.001 vs.1st quartile; IL-6 (**C**) * *p* < 0.001 vs. baseline,** *p* < 0.05 vs. baseline, **^a^** *p* < 0.001 vs. 3rd and 4th quartile, *p* < 0.05 vs. 2nd quartile, **^b^** *p* < 0.001 vs. 2nd quartile, *p* < 0.05 vs. 3rd quartile; IL-1β (**D**) * *p* < 0.001 vs. baseline, **^a^** *p* < 0.001 vs. 1st, 2nd and 3rd quartile.

Upon further analysis, we examined the impact of exercise intervention on biomarkers according to their baseline values (Figure 4). Individuals with baseline S100A8/A9 in the lowest quartile exhibited a statistically non-significant increase after exercise, while individuals in the other three quartiles exhibited a significant decrease in their S100A8/A9 levels. The magnitude of reduction was significantly greater for individuals in the higher quartile compared to those in the 2nd and 3rd quartiles (*p* < 0.001). Whereas sRAGEs were reduced across every subgroup (classified in quartiles according to baseline values), the magnitude of reduction was significantly lower in the first quartile (*p* < 0.01). Subjects in the lowest quartile of IL-6 values showed an increase in IL-6 (*p* < 0.001) after exercise while subjects in the highest quartile demonstrated a significant reduction in IL-6 values (*p* < 0.01) after exercise. No significant change was observed across 2nd and 3rd quartiles. Finally, IL-1β exhibited a great reduction in individuals with high baseline values, a modest decrease across individuals in the 2nd and 3rd quartiles and no change in individuals with low baseline values.

**Figure 5:**
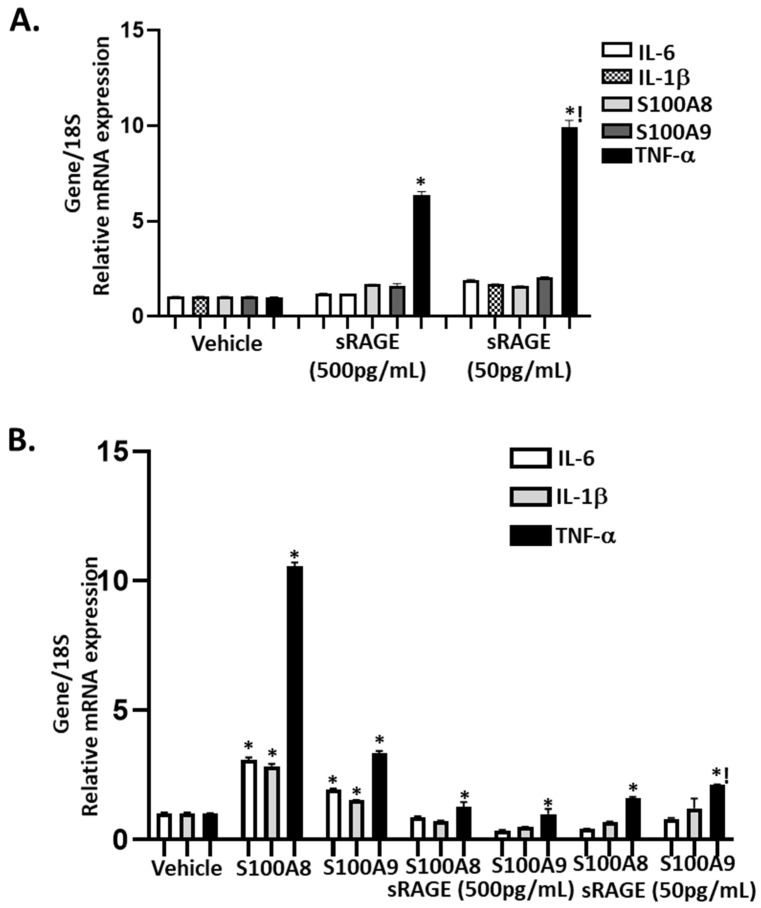
Pretreatment with sRAGEs alone or in combination with S100A8/A9 differentially regulate the expression of IL-6, IL-1β, TNF-α, and S100A8/A9. Cultured aortic vascular smooth muscle cells were pretreated (1 h) with sRAGEs (50–500 pg/mL) or vehicle (PBS) (**A**) or in combination with S100A8 or s100A9 (0.05–0.5 g/mL) (**B**). Bars are mean+SEM of gene mRNA expression. * *p* < 0.05 vs. respective vehicle; ! *p* < 0.05 vs. sRAGEs (500 pg/mL). *n* = 3 different cultures.

Next, we used ASMC cultures to determine the specific involvement of pre- and post-exercise circulating plasma levels of sRAGEs and S100A8/A9 on the expression of inflammatory markers. In the presence of circulating concentrations of 50–500 pg/mL of sRAGEs observed post and prior to the exercise intervention, respectively, of all the inflammatory markers (IL-6, IL-1β, S100A8, S100A9, and TNF-α) analyzed, only TNF-α showed a dose-dependent increase (Figure 5A). Furthermore, direct stimulation with circulating levels of S100A8/A9 induced approximately 2–11-fold increases in inflammatory marker (IL-6, IL-1β, and TNF-α) mRNA compared to vehicle control; pre-treatment with 50–500 pg/mL sRAGEs inhibited S100A8/S100A9-induced IL-6 and IL-1β and attenuated TNF-α mRNA expression (Figure 5B). Therefore, sRAGEs function as a “decoy” by binding to S100A8/S100A9 and prevent the ligation between S100A8/S100A9 and RAGE, thereby attenuating the inflammatory response.

## 4. Discussion

The main findings of our study are the significant reduction in both the pro-inflammatory molecule S100A8/A9 and sRAGEs after a four-week exercise intervention. Both results imply, in our opinion, an anti-inflammatory effect of exercise, supported by the reduction in the pivotal pro-inflammatory cytokine, IL-1β.

Our study is not only the largest one conducted to test the effects of exercise on S100A8/A9 but also the first to investigate these effects after a mid-term exercise intervention on healthy volunteers. Fagerhol et al. reported a significant increase in S100A8/A9 immediately after various types of acute strenuous exercise in a group of 52 healthy individuals [35]. However, in most subgroups, S100A8/A9 levels had returned to baseline values 1 day after exercise. Moreover, in the only group where exercise was implemented for a longer period (8 days), the intervention also included food and sleep deprivation, complicating the study results. Similarly, Mooren et al. showed an increase in S100A8/A9 in 30 healthy volunteers who underwent several types of acute strenuous exercise [36]. With the exception of a subgroup (*n* = 6) who underwent eccentric exercise, S100A8/A9 returned to baseline one day after. Both these studies highlight the pro-inflammatory environment that occurs during acute exercise but do not inform us about the potential anti-inflammatory effect mediated by the cardiovascular, hormonal, and metabolic adaptations to long-term exercise. Rochette et al. investigated the effects of a 20 min exercise bout on S100A8/A9 in a group of 12 children with idiopathic juvenile arthritis [37]. Although they observed an increase shortly after exercise, levels of S100A8/A9 were lower than baseline one day after. Only two studies to date have implemented an exercise training program to assess effects on S100A8/A9. Levitova et al. engaged 40 patients with two subtypes of axial spondylo-arthritis in a 6 month exercise training program and demonstrated a significant decrease in S100A8/A9 which was also associated with improvement in disease severity scores [38]. Similarly, Acar et al. reported a significant reduction in S100A8/A9 levels in a group of 28 rheumatoid arthritis patients who underwent an 8-week low-density exercise treatment that was also associated with improvement in disease markers [39].

Our results are in accordance with the previous studies that implemented a mid-term exercise intervention, confirming the positive effects of exercise training on S100A8/A9 levels. Furthermore, the finding that participants with higher weight and higher baseline S100A8/A9 values exhibited a greater decrease after exercise is in line with studies that investigated classic pro-inflammatory markers (mainly CRP and IL-6), where subjects with higher BMI or higher baseline values were the ones that demonstrated a significant benefit [6,9,40]. Similarly, in type II diabetic patients receiving pioglitazone, a reduction in S100A8/A9 was reported in overweight patients but not in those with normal weight [27]. The clinical implication of this finding could be that, in accordance with most medical interventions, the “sicker” individuals are likely to benefit more from exercise.

To our knowledge our study is the largest one investigating the effects of an exercise intervention on soluble RAGEs and we were able to demonstrate a significant reduction in sRAGEs after 4 weeks of military training. Moreover, the reduction observed was consistent across all subgroups of BMI categories and baseline sRAGEs values. After a thorough review of the literature, we identified four studies which examined the effects of a physical activity intervention on sRAGEs and one that measured specifically the sRAGEs isoform esRAGEs. Kotani et al. reported a significant decrease in sRAGEs levels after a 6 month exercise intervention in a group of 30 healthy sedentary Japanese (aged 65 ± 3 year) [41]. The post intervention sRAGEs levels were inversely correlated with the levels of paraoxonase1 (PON1), an antioxidative enzyme. Likewise, Farinha et al. presented a significant decrease in sRAGEs levels after 10 weeks of training, which was accompanied by an improvement in antioxidant and glycemic parameters [42]. In contrast, Choi et al. demonstrated a significant increase in sRAGEs after a 12-week exercise intervention in a group of 38 diabetic women vs. control [43]. Of note, both groups also received lifestyle advice that resulted in reduced energy intake. Furthermore, increased sRAGEs levels in diabetics have been associated with the disease state, so the results should be interpreted with caution. Sponder et al. examined the effects of 8 months of exercise training on sRAGEs in a group of 98 subjects (aged 30–65 year) with at least one CV risk factor [44]. A significant increase in sRAGEs levels was observed in the subgroup with a performance gain of >5%. Finally, Santilli et al. demonstrated a significant increase in sRAGEs after an 8-week aerobic exercise intervention in a group of 22 low-to-intermediate CV risk subjects [45]. The observed discrepancy in the response of sRAGEs after exercise possibly reflects the binary properties of sRAGEs; firstly, as a marker of increased AGEs production in cardiovascular disease and diabetes and secondly, as a decoy receptor for AGEs.

Experimental animal studies have initially established exogenous sRAGEs as an anti-inflammatory agent [46] and human studies have predominantly associated low sRAGEs levels with the state of disease [15,16,17], except for diabetics [23,24] and renal disease patients. However, other studies have reported the opposite results [18,19,20,21,22], so the role of endogenous sRAGEs remains under scrutiny. From one perspective, sRAGEs could act as a decoy for RAGE ligands, thus inhibiting their pro-inflammatory properties. Alternatively, sRAGEs may possibly reflect an extended cellular RAGEs activation (a majority of sRAGEs are produced by proteolytic cleavage of cellular receptors) and thus be a marker of disease. Importantly, the aforementioned points of view are not mutually exclusive. The complex role of sRAGEs is further supported in our study by the different pattern of change according to BMI and baseline values in comparison with S100A8/A9, a pure pro-inflammatory molecule. Consequently, in our study we do not disprove the possible anti-inflammatory properties of sRAGEs but we interpret the reduced sRAGEs post-exercise as a possible sign of diminished RAGEs expression. Importantly, the concentration of sRAGEs attained post exercise was able to inhibit/attenuate S100A8/A9-induced inflammatory marker expression in our ASMCs in vitro. Therefore, it is tempting to speculate that the circulating level (50 pg/mL) of sRAGEs realized post exercise may be sufficient to attenuate S100A8/A9 and IL-1β but not IL-6 plasma levels.

The 4-week exercise intervention did not produce significant effects on IL-6. Data in the literature concerning effects of an exercise intervention in healthy individuals on IL-6 are contradictory [8,10,11,47,48]. Our results are in line with most previous studies which failed to demonstrate a significant effect of a physical activity intervention on IL-6 even though some exhibited a significant reduction in CRP [10,11]. Moreover, the observed reduction in the subgroup of higher baseline IL-6 levels has previously been reported in a similar study [39] and complies with results with analogous studies that investigated CRP [9]. Several studies and reviews have addressed IL-6 response to exercise with the predominant theory supporting increased IL-6 production from exercising muscles, which is then termed myokine and exhibits anti-inflammatory properties. This increase probably counterbalances the expected decrease in the adipose-tissue-derived pro-inflammatory IL-6 resulting in a net anti-inflammatory effect of exercise via IL-6, despite the unaffected IL-6 serum levels. In the case of higher baseline IL-6 levels, it could be hypothesized that exercise induced a larger adipose-derived IL-6 reduction than the muscle-derived increase, resulting in the reduced post-exercise IL-6 levels in these individuals.

IL-1β possesses a pivotal role in the initiation and perpetuation of the inflammatory cascade. Apart from acute inflammatory responses it is also involved in chronic diseases, such as autoimmune diseases, type 2 diabetes, and atherosclerosis. Our findings are in accordance with the majority of the existing literature [49,50] which provides evidence for a reduction in IL-1β after exercise training, underscoring the anti-inflammatory effects of exercise.

A major strength of our study is the selection of the investigated biomarkers. S100A8/A9 and sRAGEs are emerging markers of inflammation and potentially more sensitive than those already existing. Other strengths of the study include the large sample size in comparison with previous similar studies and the composition of our sample. We have identified as a potential limitation in previous studies the heterogeneity of their sample, as chronic low-grade inflammation varies significantly among apparently healthy individuals and is strongly associated with increasing age [51] and body weight [52]. With regard to sex, although female sex is not associated with low-grade inflammation per se, women exhibit a larger increase in inflammatory markers with increasing body fat in comparison to men [53]. Moreover, participants in many studies, although free from overt CVD, suffered from diseases (dyslipidemia, hypertension, diabetes) or were on medication (statins, non-steroid anti-inflammatory drugs) that could critically affect inflammatory status. In our study, we recruited young healthy males, thus minimizing the potential variability of low-grade inflammation driven by sex and age differences or co-morbidities.

Potential limitations of our study include the lack of a control group, as well as the lack of data concerning changes in anthropometric measures (BMI) after training. Finally, due to the nature of the sample (army recruits), the intensity of physical exercise could not be individualized.

## 5. Conclusions

A 4-week military training resulted in a significant reduction in the pro-inflammatory S100A8/A9 complex. The levels of sRAGEs were also significantly reduced and were interpreted as a sign of diminished RAGEs axis activation. Our study has the significance of being the first to demonstrate a positive effect of exercise on the above-mentioned markers in apparently healthy individuals. Larger placebo-controlled trials are warranted to verify these results and expand the use of such biomarkers in clinical practice to monitor the effect of exercise on inflammation.

## Figures and Tables

**Table 1:** Anthropometric characteristics of the study population.

**Age (years)**	21.07 ± 1.63
**Weight (kg)**	78.12 ± 13.47
**Height (cm)**	180.13 ± 6.3
**BMI (kg/m²)**	24.06 ± 3.87
**Systolic BP (mm Hg)**	125.88 ± 13.88
**Diastolic BP (mm Hg)**	77.29 ± 9.19

**Table 2:** Biomarkers’ levels before and after exercise according to BMI.

	BMI (Median Value = 24.035)		
	Below Median	Above Median	
**sRAGEs (pg/mL)**			
**Day 0**	431.8 ± 138.5	363.67 ± 153.48	*p* = 0.003
**Day 28**	240.93 ± 179.66	199.36 ± 181.77	*p* = 0.14
	*p* < 0.001	*p* < 0.001	
**S100A8/A9 (ng/mL)**			
**Day 0**	606.7 ± 232.5	645.9 ± 240.9	*p* = 0.287
**Day 28**	506.9 ± 228.8	434.4 ± 176.7	*p* = 0.023
	*p* = 0.002	*p* < 0.001	

BMI Categories according to WHO Classification: <18.5 underweight, 18.5–25 normal weight, 25–30 overweight, >30 obese.

**Table 3:** Biomarkers’ levels before and after exercise across BMI categories.

	BMI			
	Underweight (*n* = 6)	Normal Weight (*n* = 100)	Overweight (*n* = 45)	Obese (*n* = 15)
**sRAGEs (pg/mL)**				
**Day 0**	528.3 ± 115.4	415.4 ± 140.8 ^a^	362.47 ± 153.2 ^a,b^	333.5 ± 165.3 ^a,b^
**Day 28**	278 ± 188.4	234.1 ± 175.8	210.7 ± 206.9	132.4 ± 103.1 ^a,b^
	*p* = 0.016	*p* < 0.001	*p* < 0.001	*p* < 0.001
**S100A8/A9 (ng/mL)**				
**Day 0**	723.2 ± 165.8	586.3 ± 226.2 ^a^	700.7 ± 256.8 ^b^	627.5 ± 218.9
**Day 28**	617.1 ± 283.1	487.2 ± 218.6	444.3 ± 181.5 ^a^	379.9 ± 112.9 ^b^
	*p* = 0.346	*p* < 0.001	*p* < 0.001	*p* < 0.01

BMI Categories according to WHO Classification: <18.5 underweight, 18.5–25 normal weight, 25–30 overweight, >30 obese. ^a^ Statistically significant difference compared to underweight. ^b^ Statistically significant difference compared to normal weight.

## Data Availability

All data is presented in the manuscript. As the data are drawn from a military population, any additional details will only be made available upon a formal specific request made to the corresponding author who will seek approval from the relevant agencies. A formal request will not infer approval.

## Abbreviations

AGEs	Advanced Glycation End Products
ANOVA	Analysis of Variance
ASMCs	Aortic smooth muscle cells
BMI	Body Mass Index
CV	Cardiovascular
CVD	Cardiovascular Disease
CRP	C-Reactive Protein
DAMP	Damage-associated molecular pattern
EDTA	Ethylenediamine Tetra-acetic Acid
ELISA	Enzyme-linked Immunosorbent Assay
esRAGEs	Endogenous secreted Receptor for Advanced Glycation End Products
HMGB-1	High Mobility Group Box-1
IL-1β	Interleukin-1β
IL-6	Interleukin-6
PBS	Phosphate buffered saline
PON1	Paraoxonase 1
RAGEs	Receptor for Advanced Glycation End Products
sRAGEs	Soluble Receptor for Advanced Glycation End Products
TLR4	Toll-Like Receptor 4
WHO	World Health Organization
